# Expression, purification and characterization of the *Lily symptomless virus *coat protein from Lanzhou Isolate

**DOI:** 10.1186/1743-422X-7-34

**Published:** 2010-02-10

**Authors:** Ruoyu Wang, Guangpeng Wang, Qi Zhao, Yu Zhang, Lizhe An, Yun Wang

**Affiliations:** 1 Cold and Arid Regions Environmental and Engineering Research Institute, Chinese Academy of Sciences, Lanzhou 730000, China; 2 Research Center for Eco-Environmental Sciences, Chinese Academy of Sciences, Beijing 100085, China; 3 Key Laboratory of Arid and Grassland Agroecology of Ministry of Education, School of Life Sciences, Lanzhou University, Lanzhou 730000, China; 4 Department of Bacteriology, Graduate School of Medical Science, Kanazawa University, Kanazawa, Ishikawa 920-8640, Japan

## Abstract

**Background:**

Lily symptomless virus (LSV) is widespread in many countries where lily are grown or planted, and causes severe economic losses in terms of quantity and quality of flower and bulb production. To study the structure-function relationship of coat protein (CP) of LSV, to investigate antigenic relationships between coat protein subunits or intact virons, and to prepare specific antibodies against LSV, substantial amounts of CP protein are needed.

**Results:**

Thus, full-length cDNA of LSV coat protein was synthesized and amplified by RT-PCR from RNA isolated from LSV Lanzhou isolate. The extended 33.6 kDa CP was cloned and expressed prokaryoticly and then purified by Ni-ion affinity chromatography. Its identity and antigenicity of recombinant CP were identified on Western-blotting by using the prepared anti-LSV antibodies.

**Conclusions:**

The results indicate that fusion CP maintains its native antigenicity and specificity, providing a good source of antigen in preparation of LSV related antibodies. Detailed structural analysis of a pure recombinant CP should allow a better understanding of its role in cell attachment and LSV tropism. This investigation to LSV should provide some specific antibodies and aid to development a detection system for LSV diagnostics and epidemiologic surveys.

## Background

Lanzhou lily (*L. davidii Duch.var*) is an important bulb edible crop which mostly distributes in middle area of Gansu province in China. Virus infection caused serious reduction in production of Lanzhou lily and other economic corps in recent years [1,2]. *Lily symptomless virus *(LSV; family, Genus *Carlavirus*, species) is the most prevalent virus infecting Lanzhou lily [2], and it has been reported in USA, Europe, Australia and Asia [3-7]. It is also one of the most harmful viruses of lilies that causes severe losses in terms of quantity as well as quality of bulb and flower production[8]. The host range of LSV is mostly distributed in genus *Lilium*, however, in one case reported in *Alstroemeria*[9]. The observed abnormalities such as growth reduction, smaller flowers and lower bulb yield can be caused by combined infection with LSV and cucumber mosaic virus (CMV) [8] which threatens the yield and commercial production of lily plants.

LSV contains a filamentous viral particle, 640 nm in length and 17-18 nm in diameter. The genomic RNA of LSV is constituted of 8,394 nucleotides (excluding the poly (A) tail) and contains six open reading frames (ORFs) coding for proteins of Mr 220 kDa (1,948 aa), 25 kDa (228 aa), 12 kDa (106 aa), 7 kDa (64 aa), 32 kDa (291 aa) and 16 kDa (140 aa) from the 5' to 3' end respectively, composed of monopartite, single-stranded, plus sense RNA molecules. The ORF5 (7140-8015 nts) encodes a CP of 291 aa and genomic RNA of LSV is encapsidated by the single type of CP with a Mr of 32 kDa [10,11]. The 3' terminal of carlavius group is linked with a poly (A) tail [12,13].

In this study, we cloned, expressed and purified a complete coat protein of LSV and prepared polyclonal antibodies for the recombinant protein. As a result this experiment was to demonstrate the antigenicity of recombinant LSV CP and to aid to further development of an efficient immunoassay for LSV diagnostics and epidemiologic surveys for Lanzhou lily.

## Results and discussion

### RT-PCR and construction of expression vector

The expected 900 bp cDNA encoding LSV CP was amplified by RT-PCR from isolated RNA. The constructed plasmid for expression has been checked by gene sequencing.

### Expression, Purification and identification

**Figure 1:**
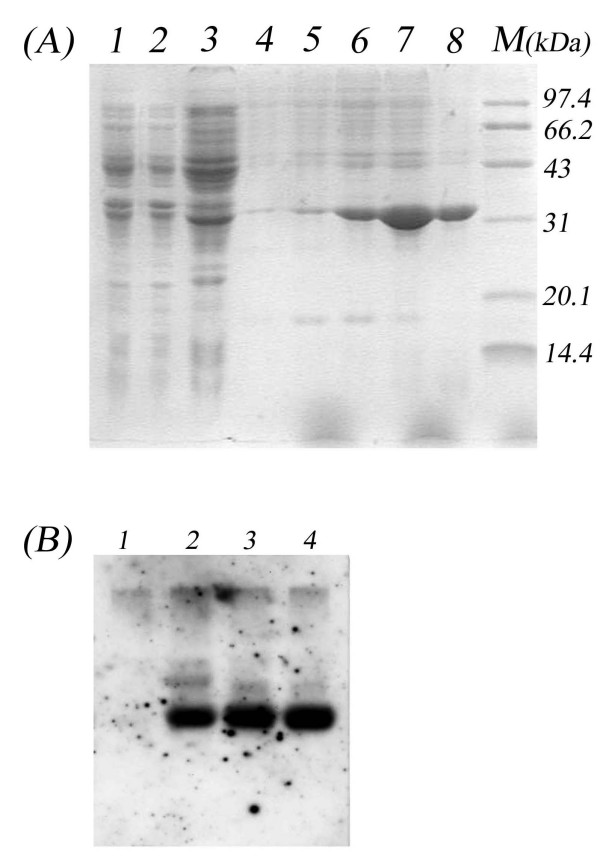
**Expression and purification of LSV coat protein**. A, Expression and purification of LSV CP. 15% SDS-PAGE of cultures uninduced E. coli BL21 (Lane 1), pET28 transformed E. coli BL21 as negative control (Lane 2), IPTG induced (Lane 3). Each sample of every 2 ml intervals of all eluted fractions (lane 4 to 8), all of samples were cultured in 1 mM IPTG contained LB at 37°C. Lane M, protein molecular weight markers. B, Western blotting using rabbit anti-LSV (intact virus preparations) as first antibody. Lane 1, pET28a transformed E. coli BL21 as negative control; 2,3 pLCP319 transformed strains; 4, purified Coat Protein of LSV lanzhou isolate.

Colonies presenting the strongest amplicon of the expected size were chosen for small-scale expression trials to determine the best harvesting time. Shows one such experiment, in which maximum expression was attained at 5-6 h after IPTG induction. By 1 h after induction, an additional remarkable band of approximately 34.5 kDa could be detected among the endogenous bacterial proteins in Coomassie blue stained gel, becoming increasingly evident during the following 6 h. The purified protein was subjected to SDS-PAGE and a western blotting was carried out with anti-LSV polyclonal antibody. Specific blotting bands were detected at the corresponding positions (Fig.1a and 1b). Apparently molecular mass of the purified protein was about 34.5 kDa as expected. These results demonstrated an intact immunologic reactivity of the recombinant CP of LSV. The yield was about 8.32 mg purified LSV CP from 1000 ml of bacterial culture after a single step of Ni^2+ ^affinity chromatography. The recovery of LSV CP was about 26%. A NcoI site containing a ATG codon was used so that the N-terminal of the recombinant protein without surplus amino residues was finally obtained. However a downstream hexahistidine stretch of pET28a(+) was taken advantage of and a 13-amino acid residues C-terminal extension was followed the native LSV coat protein. Recombinant LSV CP obtained in this way has an only 13-amino acid extension in C-terminal.

### MALDI-ToF MS and phylogenetic analysis

**Figure 2:**
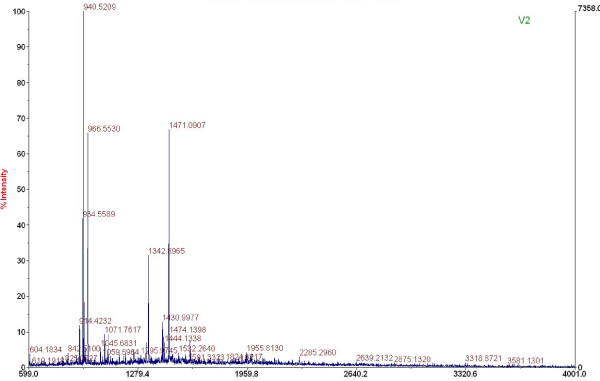
**MALDI-TOF mass spectrum of the tryptic digested LSV coat protein**.

The MALDI-ToF mass spectrum of tryptic digest of the gel band of His-CP was shown in Fig.2a. The achieved peptide masses were searched against NCBInr database without any species limitation and with peptide mass tolerance of ± 0.1 Da by using the Mascot search engine. The first candidate protein, with a score of 192, was coat protein of LSV isolated from India (Genbank CAE51028). The second to the 23th candidate proteins had scores from 82 to 178, those were all coat proteins of various isolates of LSV, and protein scores greater than 67 were significant (p < 0.05). 15 of 28 mass values of searched peptide fragments were matched with tryptic digested peptides of LSV CP, and the sequence coverage was 58%.

**Figure 3:**
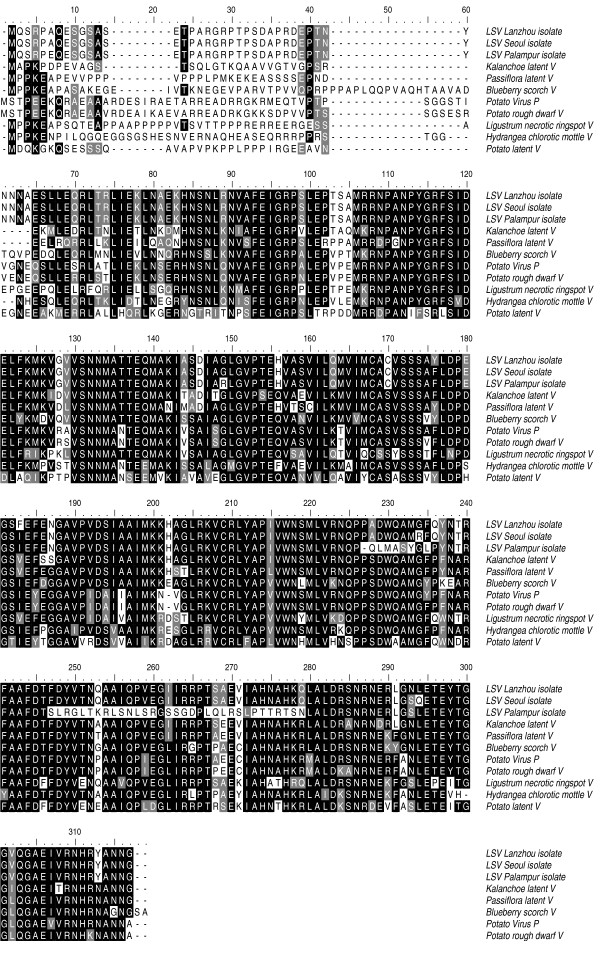
**Alignment of the amino acid sequence of coat protein of different LSV isolates and other carlavirus**. Shading with black color indicated that the amino acid sequences were identical, whereas shading with grey color indicated that the amino acid sequence were similar

After BLAST searches against protein sequence databases in GenBank, significant levels (98-80%) amino acid sequences identity among CP of LSV and some other virus was clearly revealed. A high level of identity was found with some LSV isolates which had been reported ([11,14] and considerable similarities were found with *Kalanchoe latent virus *[Genbank:AAO92328], *Passiflora latent carlavirus *[Genbank: YP_717537], *Blueberry scorch virus *[Genbank: AAY18407], *Potato Virus P *[Genbank: ABF59717], *Potato rough dwarf virus *[Genbank: ABG21368], *Ligustrum necrotic ringspot virus *[Genbank:YP_002985640], *Potato latent virus *[Genbank: YP_002302561] (Fig.3). Multiple alignments of the CP sequences among the above-mentioned virus revealed an overall homology among isolates.

### Antigenicity analysis of recombinant CP

**Figure 4:**
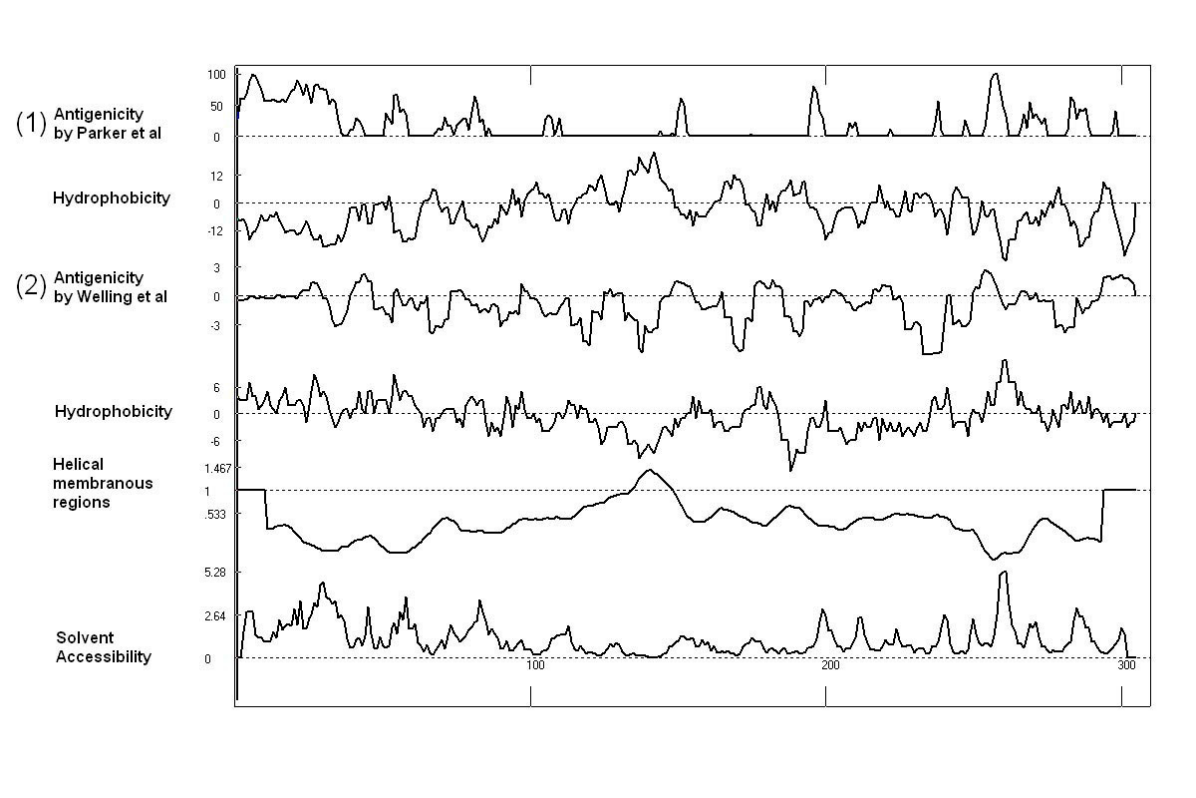
**Antigenicity analysis of His-tagged LSV CP**. (1) Antigenicity analysis with the method as described by Parker et al and (2) Antigenicity analysis with the method as described by Welling et al.

The antigenicity based on primary structure and partly putative secondary structure was analyzed by software ANTHEPROT V 4.3c (Fig.4). There are some obvious differences in antigenicity between two analysis which derived by these two methods described by Parker et al. and Welling et al. respectively. The former shown a stronger antigenicity of from N terminal to 100 aa of the LSV CP, although from 260 aa to 300 aa of the LSV CP shown a relative stronger antigenicity also, while the later indicate that antigenicity almost distributed evenly of the whole protein sequence of LSV CP. From the prediction of secondary structures, the LCP contains 38% α-Helix, 10% β-Sheet, 4% β-Turn and 48% Coil structures. By analysis of antigenicity, peptides 1-37 (MESRPAQESGSASETPARGRPTPSDAPRDEPTNYNNN), 50-59(IEKLNAEKHN), 67-87(FEIGRPSLEPTSAMRRNPANP),194-201(MLVRNQPP),253-275(QLALDRSNRNERLGNLETEYTGG), 281-290(IVRNHRYANN) of all 304 amino acid residues are located in antigenic determinants dense regions. These peptides mostly belong to Coil structures. All of these antigenic determinants dense regions sum up 109 amino acid residues, account for 36% of the whole protein. It can be predicted that most antibodies contained in the polyclonal antibodies are specific against antigenic determinants presented among these peptides of the protein. After completion of renaturation, tertiary structure conformation of recombinant LCP should be farthest similar to that of native CP, as a consequence, it will keep most of antigenicities of native one. An intact viral particle of LSV is assembled by a viral RNA molecule and about 1800 CP subunits. Intact virus contains total, or at least most antigenicities of coat protein, but part of these antigenic determinants are blocked due to hindrance from space conformation among subunits, whereas some new antigenic determinants which are derived from more complex structure of aggregated subunits are brought about. Thus, cross-antigenicities occurred between intact virus and individual subunits are those antigenic determinants which are not blocked by hindrance from space conformation.

### Charaterzition of Antibodies

By double immunodiffusion test, titres of anti His-CP of LSV antibodies can reach to a 1/1024 dilution, this result also verified the strong specificity to components of native LSV in respect with the antibody. By bleeding and separation of antiserum and DEAE cellulose column purification, anti-LSV CP IgG (ALCP) was isolated.

**Figure 5:**
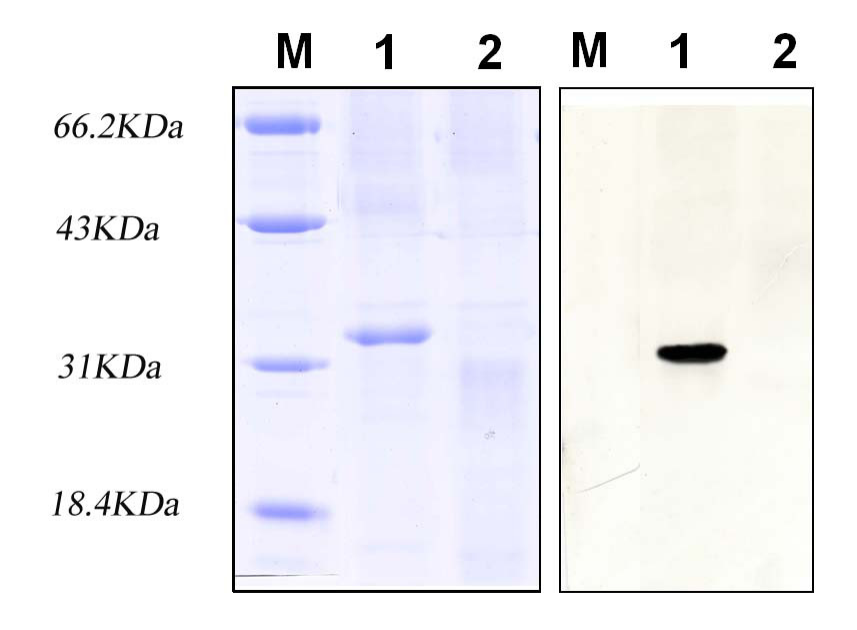
**Antibody characterization**. Characterization analysis of polyclonal Antibodies against His-tagged LSV CP with Western blotting using native coat protein of LSV as antigen.

A 34.5 kDa specific blotting band was detected at the corresponding position by Western blotting (Fig.5). The results showed that the native coat protein of LSV also reacted positively to ALCP raised against recombinant His-tagged CP. Furthermore, both results of double immunodiffusion and Western blotting demonstrate that ALCP does have a high specificity to native and denatured CP of LSV. As a result, antigenicity of recombinant LSV CP has also been identified. These results seem to have some contradictions with conclusion that intact LSV and pyrrolidine degraded LSV have very few antigenic determinants in common or none at all [15]. To our knowledge, LSV degraded by pyrrolidine are small fragments or even subunits for coat protein. Here we take advantage of ultrasonic degraded fragments of intact LSV as antigenic components in double immunodiffusion. However, there aren't substantial differences between pyrrolidine degraded and ultrasonic degraded LSV. It can be demonstrated that ALCP is able to react satisfyingly with both of intact virus and degraded viral fragments. As an excellent result, the recombinant LSV CP can undertake an antigenic reagent for providing valuable resources for LSV diagnostics and epidemiologic surveys.

## Conclusions

The results indicate that fusion CP maintains its native antigenicity and specificity, providing a good source of antigen in preparation of LSV related antibodies. Detailed structural analysis of a pure recombinant CP should allow a better understanding of its role in cell attachment and LSV tropism. This investigation to LSV should provide some specific antibodies and aid to development a detection system for LSV diagnostics and epidemiologic surveys.

## Methods

### Strains, plasmids, and enzymes

The *E. coli *strains DH5α and BL21(DE3) were used for cloning experiments and protein expressions, respectively. Both strains were purchased from Invitrogen (Invitrogen, Carlsbad, CA, USA). The plasmid pUCm-T vector was used for cloning and amplification of LSV CP cDNA and the plasmid pET28a(+) (Novagen, Darmstadt, Germany) was used for protein expression. The plasmids were from Sangon and Novagen, respectively. Restriction enzymes, MMLV reverse transcriptase Taq DNA polymerase, and T4 ligase were purchased from Promega and used according to supplier's recommendations.

### Sample preparation for DAS-ELISA

Naturally infected Lanzhou Lily were sample in Xiguoyuan (Lanzhou, China) then tested by DAS-ELISA kit (Agdia, USA) according to manufacturer's instruction. 20 g of LSV positive tissue of leaves was crashed and put into liquid nitrogen, 40 ml of Extraction Buffer and 30 ml of chloroform was subsequently added. By completing homogenization, the mixture was centrifuged at 5000 g for 15 min. When supernatant was filtered by a sterile filter, 8% (w/v) of PEG 6000 was slowly added to the preparation and then stored overnight at 4°C. Then a centrifugation at 5000 g for 30 min was carried out and the precipitate was collected and resuspanded in 1 ml pH7.2 0.1 mol/L PB.

### RNA isolation, Reverse transcriptase-PCR and cDNA cloning

The viral RNA of LSV was extracted with Trizol reagent (Invitrogen) according to the manufacturer's instruction. The first strand cDNA was synthesized by the MMLV reverse transcriptase with the Oligo (dT)-18 primer as the protocol described by 15 Nie and Singh (2000). Two primers LCP1 (5'-CACCATGGAATCAAGACCAGCAC-3') and LCP2 (5'-ATAAGCTTTCCATAATTTGCGTATCG-3') were designed according to DQ531052 of NCBI (Wang et al., 2007). The sequences underlined are the recognition sites of the restriction enzymes NcoI and HindIII. Takara Ex taq (Takara, Japan) DNA polymerase was used in amplification (94°C for 45 s, 57°C for 45 s and 72°C for 1 min, 35 cycles). The amplicon was introduced into the pUCm-T vector (BBI) and named pLCP312, analyzing and quantification have been carried out in agarose gel electrophoresis and in comparison with the standard molecular weight marker.

#### Construction of CP expression plasmid

The plasmid pLCP312 was digested with NcoI and HindIII restriction enzymes and ligated into NcoI/HindIII-digested pET28a(+) vector for 1 h at 16°C through a standard T4 DNA ligase procedure. The obtained plasmid pLCP319 which carrying a His-tagged *cp *gene were transferred to *E. coli *BL21(plys) and selected in LB plates supplemented with 50 μg/ml of kanamycin.

### Expression and Purification

The expression plasmid, pLCP319, containing the expected sequence was used to transform competent *E. coli *BL21(DE3). The bacterial cells were cultured in LB broth contained 50 μg/ml kanamycin and shaken overnight at 37°C. Then culture was transferred into 50 ml fresh LB medium and grown at 37°C with vigorous shaking. The following induction and identification of isopropyl-1-thio-β-d-galactoside (IPTG) induced CP was munupulated with standard procedure according to manufacture instructions. The lysate of cultured bacteria was clarified at 10,000 g for 10 min at 4°C and both the soluble fraction and the pellet containing the insoluble fraction (inclusion bodies) were analyzed by SDS-PAGE. The pellet of inclusion bodies was washed with 5 ml 0.01 M pH 8.0 Tris-HCl containing 0.1 M sodium phosphate buffer, and 2 M urea, and incubated at room temperature for 30 min. Then the purified inclusion bodies were solubilized in denaturing buffer (0.01 M Tris-HCl, 0.1 M sodium phosphate buffer, and 8 M urea, pH 8.0), and incubated on ice for 1 h. After centrifugation at 12,000 g for 30 min, the supernatant was collected and the protein concentration was determined.

The supernatant was filtered by a 0.45 μm filter and then applied to an 5 ml His-Trap HP pre-packed column (Amersham Biosciences) using an ÄKTA basic 100 Purification System (GE Healthcare, USA) followed by washing the column with Wash Buffer (8 M urea, 25 mM imidazole in 10 mM Tris-HCl pH 8.0), the protein of interest was then eluted with Elution buffer (8 M urea 250 mM imidazole in 10 mM Tris-HCl pH 8.0). The purified protein solution was quantified as described of Lowry et al (1951) in respect to standardization with bovine serum albumin, and estimated by its molar extinction coefficient at 280 nm.

### SDS-PAGE and Western-blotting analysis

Samples from induced bacterial culture and purified His-LSV CP were resolved on 15% SDS-PAGE and visualized by Coomassie blue R250 staining and then electroblotted onto a nitrocellulose membrane (Millipore) using a Semi-Dry Transfer System (Bio-Rad) at 100 mA for 1 h. Non-specific protein binding was blocked by incubating the membrane in PBST (PBS containing 0.05% Tween 20) and 5% slim milk at room temperature overnight. The blot was incubated 1 h at 37°C in PBST containing rabbit anti-LSV (intact virus preparations) polyclonal antibodies (1:200). Finishing washing with PBST, the membrane was incubated with digoxigenin-dUTP-linked goat anti-rabbit IgG for 90 min. Signal detection was performed with anti-digoxigenin alkaline phosphatase conjugated Fab fragments and CSPD-star ready-to-use From Roche Molecular Biochemicals (Indianapolis, IN, USA). All experiments were performed at least three times with similar results.

### MALDI-ToF MS, Database searching for His-CP

His-CP of LSV containing band was cut from the gel and destained overnight with a solution of 50 mM ammonium bicarbonate, 40% ethanol. The protein was digested in gel with trypsin (Promega). Peptide mass mapping was performed by matrix assisted laser desorption-ionisation/time-of-flight mass spectrometry (MALDI-TOF-MS) using a Proteomics System I (ABI, USA). The peptide map was acquired in reflectron positive-ion mode with delayed extraction at a mass range of 600-4000 Da. Peptide mass fingerprint (PMF) and MS/MS data from MALDI-TOF-MS were analyzed by searching against an NCBInr database using GPS (Matrix Science, London) search software. The relative molecular mass (Mr) range: 15-80 kD (1 Da mass tolerance), a minimum of four peptides must be matched and a maximum of one missed tryptic cleavage point.

### Production of Anti-LSV CP polyclonal antibodies

By using prepared His-tagged LSV CP to immunize rabbits, antisera were produced. Immunization was carried out by subcutaneously injecting several sites into rabbits with 1 mg of purified His-LSV CP, emulsified with an equal amount of Freund's complete adjuvant (Sigma, USA). Boost was followed in the same dosage with Freund's incomplete adjuvant (Sigma, USA) injecting subcutaneously or intramuscularly at every two weeks for 4 times. 10 days after last injection, titres of blood were determinated by a double immunodiffusion, 0.1 mg/ml of purified LSV (Lanzhou isolate) was employed as antigen in this test. When the titres reached to more than 1/64 dilution, terminal bleeding and isolating the antisera were performed. Polyclonal antibodies were purified by precipitation with ammonium sulphate and passaged through a column of DEAE cellulose, and designated as ALCP.

## Competing interests

The authors declare that they have no competing interests.

## Authors' contributions

RW carried out the molecular genetic studies, immunoassays and drafted the manuscript. YZ, GW and QZ participated in the sample preparations. YW participated in the design of the study. LA conceived of the study, and participated in its design and coordination and helped to draft the manuscript. All authors read and approved the final manuscript.
